# The Megameatus, Intact Prepuce Variant of Hypospadias: Use of the Inframeatal Vascularized Flap for Surgical Correction

**DOI:** 10.3389/fped.2018.00055

**Published:** 2018-03-14

**Authors:** Marc Cendron

**Affiliations:** ^1^Department of Urology, Boston Children’s Hospital, Harvard Medical School, Boston, MA, United States

**Keywords:** hypospadias, megameatus, intact prepuce, surgical repair, Mathieu technique

## Abstract

**Introduction:**

The megameatus intact prepuce (MIP) variant of hypospadias is a rare variant of hypospadias that is diagnosed either early at the time of circumcision or later as the foreskin is retracted. The true incidence of the anomaly is difficult to determine precisely as some patient never come to medical attention but is felt to under 5% of all cases of hypospadias. The purposes of this study are to review the embryology and clinical findings of MIP and then, in light of a personal experience, present a series of patients evaluated for MIP who were treated with a modification of the Mathieu technique.

**Materials and methods:**

A PubMed search of all articles in the MIP variant of hypospadias was carried out followed by an exhaustive review of the literature. The charts of all patients evaluated and treated at Boston Children’s Hospital by MC between 2007 and 2017 were reviewed retrospectively. The patients were divided into two groups: those who underwent the standard procedure and those who underwent a repair using a modification of the Mathieu procedure using an inframeatal flap.

**Results:**

The embryologic explanation of the MIP variant is not clear but failure of the distal, glanular portion of the urethra to tubularize results in spectrum of abnormality characterized by a deep glanular groove and an abnormal opening of the urethra anywhere from the mid-glans to a subcoronal location. Surgical repair is complicated by a wide distal urethra which may be injured if not properly identified. Overall good outcomes were noted with one patient experiencing a urethra cutaneous fistula in the first group and one patient having a mild glans dehiscence in the second.

**Conclusion:**

The MIP variant of hypospadias is a rare variant of hypospadias that presents as a spectrum of urethral anomaly. Surgical repair may not always be necessary but if surgical repair is carried out, the Mathieu technique modification may offer better anatomic delineation of the urethra and will provide an extra layer of tissue to cover the reconstructed urethra. Low complication rates should be expected with adequate functional outcome such as a normal urinary stream. In addition, criteria for selecting patients for surgical repair are provided.

## Introduction

The megameatus intact prepuce (MIP) variant of hypospadias is a rare variant of glanular hypospadias, occurring in approximately 3–6% of all hypospadias repair although its exact incidence is unknown given the fact that a number of patients with MIP may not be identified, come to medical attention or the urethral anomaly is not felt to be clinically significant. In the series presented herein, a retrospective accounting shows that 25 of the 481 hypospadias (5%) carried out over the past 10 years had the diagnosis of MIP. Seven more cases were felt to be mild and asymptomatic. Surgery was not recommended in these cases as they may not be associated with functional issues.

Very few articles have focused on this form of hypospadias variant and its management, the first description being provided by Juskiewenski et al. in 1983 (1). 6 years later Duckett and Keating described the congenital defect in detail and put forth the “pyramid procedure” as a surgical treatment for the patient with MIP (2). Since, a handful of retrospective case-studies have been published (3–6).

The purpose of this article is to provide a brief discussion of the embryology, describe the anatomic findings of MIP hypospadias variant and then review the surgical treatment in light of a personal series using a modification of the Mathieu procedure for the repair.

## Embryology

Elaboration of the male urethra occurs between the fifth and sixth week of gestation when the genital tubercle enlarges and differentiates. Ventrally, the urethral groove deepens and the urethral folds located on either side of the grove enlarge. These folds then progressively fuse from the base of the phallus toward the glans. It has been theorized that hypospadias results from incomplete fusion of the urethral folds resulting in an incomplete urethra and incomplete or hooded foreskin. In the MIP variant of hypospadias, formation of the prepuce is normal but the distal portion of the urethra remains open. Overcanalization or persistent splitting of the distal, ventral urethra may cause disunion of the glans and formation of an abnormally large meatus associated with a deep glans cleft (2, 3). Interestingly, the glanular urethra seems to widen at the level of the corona but will taper back down to a normal caliber in the distal penile urethra.

The prepuce forms normally and is usually intact. Some authors have reported MIP variant of hypospadias as the result of a possible consequence of neonatal circumcision injury but that notion should be dispelled as noted by Peretz and Westreich (7).

## Clinical Findings

Megameatus intact prepuce hypospadias variant is found either late in life in non-circumcised boys at the time of retraction of the prepuce or at the time of neonatal circumcision. Most, if not all patients appear to have a normal urinary stream. MIP variant of the hypospadias can be associated with a large variation in the appearance of the urethral meatus. Some patients will have a mildly dilated meatal opening; whereas some may present with a large fish mouth opening which may expand down or just below the coronal margin. The patients who present with the more enlarged forms of the meatus may present with a splayed urinary stream. As mentioned earlier, the prepuce is normal. Curvature of the penis is not present. No other urologic anomalies have been associated with MIP variant of hypospadias and no radiologic evaluation is needed in the absence of any other symptomatology.

Megameatus intact prepuce variant of hypospadias may in fact not be associated with any functional issues. No reports were found documenting penile curvature. Therefore artificial erection at the time of repair is not recommended. Furthermore, some patients display a slightly enlarged urethral opening which may not require intervention.

In the newborn, with a normal foreskin, the hypospadiac meatus may only come to light during or after circumcision. This may be the cause of undue anxiety on the part of the practitioner as the foreskin is not needed for the subsequent repair. The circumcision can thus be carried out with no guilt.

## Surgical Management

Patients diagnosed with MIP variant of hypospadias should be referred to a pediatric urologist before age 6 months. Surgical repair may be offered for those patients with a large fish mouth or blunderbuss appearing meatus that opens close or at the coronal margin. Proper recognition of the defect will help dictate the surgical approach. In general, the urethral plate will be irregular and may extend laterally at the level of the coronal margin making the dissection more difficult. If the lateral extent of the distal urethral is not recognized, injury to the urethra at the time of repair may make reconstruction of the urethra more problematic.

If MIP variant hypospadias has been identified and surgical treatment is considered, surgery should be carried out between ages 6 and 18 months (similarly to other forms of hypospadias) under general anesthesia with either a penile or caudal regional block.

The incision is marked out and the incision is delineated. In cases where the foreskin has not been removed, degloving of the penis on the ventral aspect can be carried out after retracting the foreskin. In cases where circumcision has occurred, partial degloving can be carried out ventrally after carefully circumscribing the meatus. Holding sutures placed laterally and below the meatus will allow for carful mobilization of the urethra. Two vertical incisions are then made in the glans parallel to the urethral plate. This will initiate dissection of the glans wings preserving the urethral mucosa and sub mucosa. Local injection of a solution of lidocaine between the glans, urethral plate, and corpus cavernosum may facilitate identification of the planes between these three contiguous structures.

Once the urethral plate has freed up from the glans, edges of the mucosa may need to be trimmed and then brought back together in the midline over an 8 F urethral catheter, using fine absorbable suture material. Interrupted subcuticular stitches are preferred. A layer of subcutaneous tissue can then be brought over the reconstructed urethra in order to provide an interposed vascularized layer of tissue (4, 8). Glans wings are then brought back over the urethra and the urethra meatus is matured. Care should be given to avoid narrowing of the meatus excessively in all attempts to bring it as close to the tip of the penis as possible it can only increase the risk of meatal stenosis.

**Figure 1:**
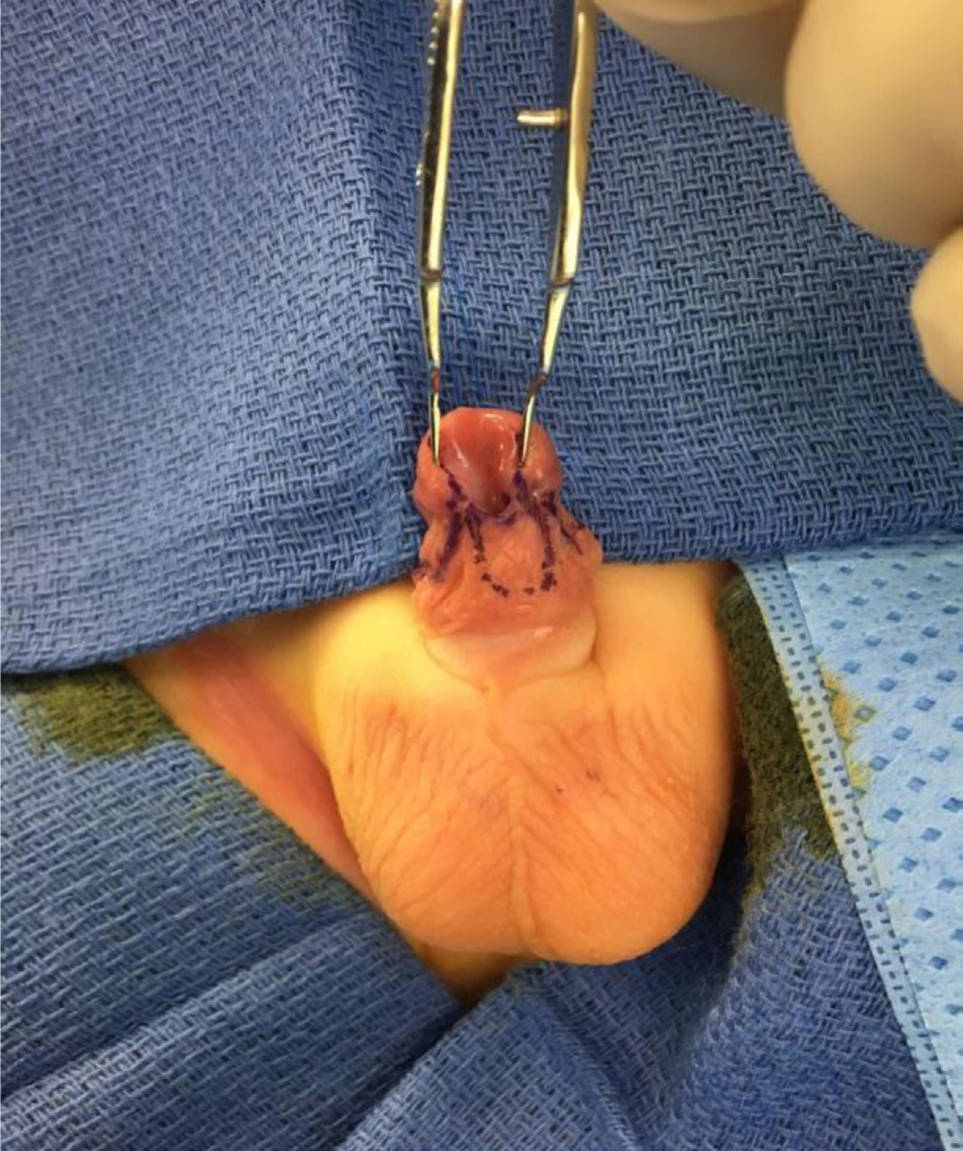
Preoperative outlining of the incision. Note that the glans is splayed and the urethral groove is deep. The infracoronal flap has been measured to match up the length of the urethral plate.

**Figure 2:**
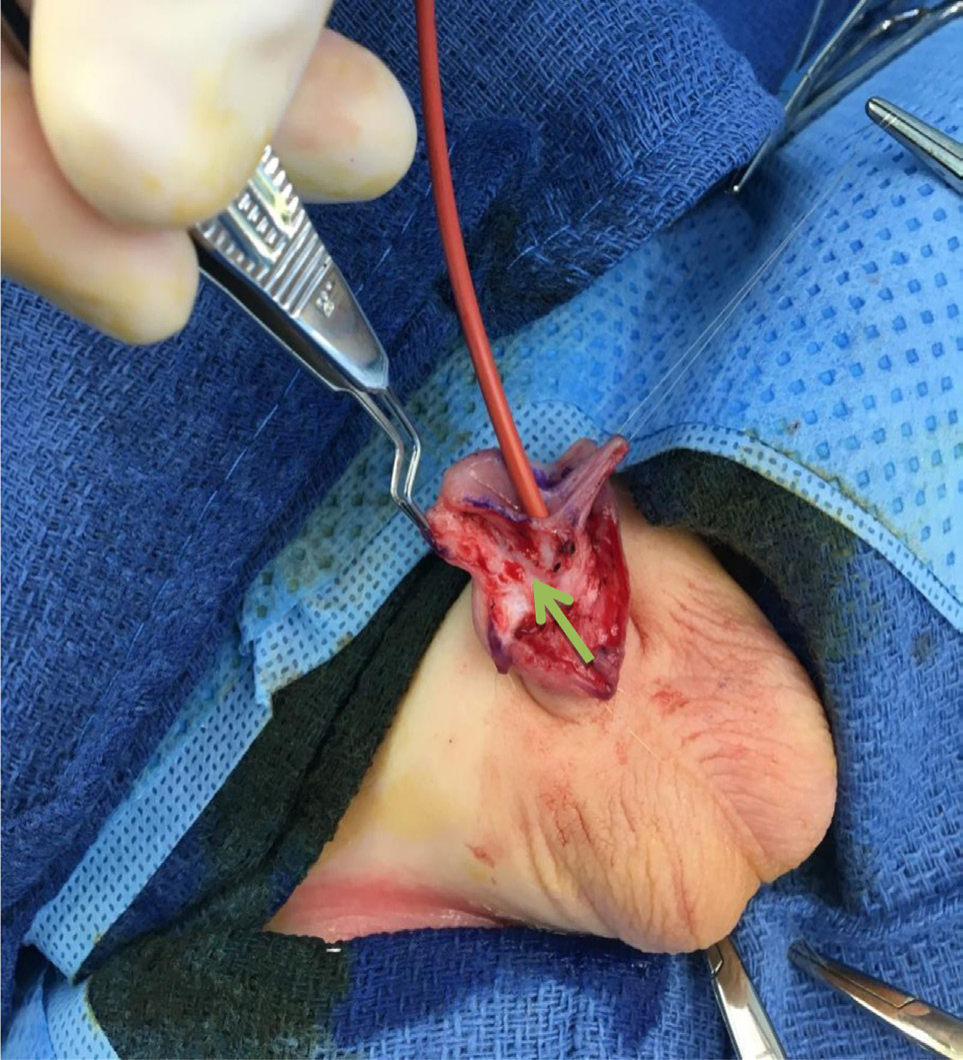
Intraoperative view of the urethral plate dissection. Note the clear delineation of the lateral extension of the urethral plate (green arrow). A traction suture on the inframeatal flap allows for identification of the tissue planes between urethra and glans.

Recently, we have adapted the principle of the Mathieu procedure to the repair of MIP variant of hypospadias to enable better visualization and mobilization of the urethra and to provide a healthy flap of vascularized subcutaneous tissue for urethral coverage (9). Prior to dissection of the urethra plate, an inframeatal flap of penile skin is marked out to the length of the urethral plate. It is then incised and dissected up with fine iris scissors (Figure 1). A traction suture is placed on the inferior aspect of the flap in order to facilitate traction and dissection. Once the flap is freed up, the urethra can then be carefully mobilized and the lateral extent of the plate can be well identified on either side. This in turn allows for a careful dissection of the glans wings and prevents an inadvertent injury to the urethra (Figure 2).

**Figure 3:**
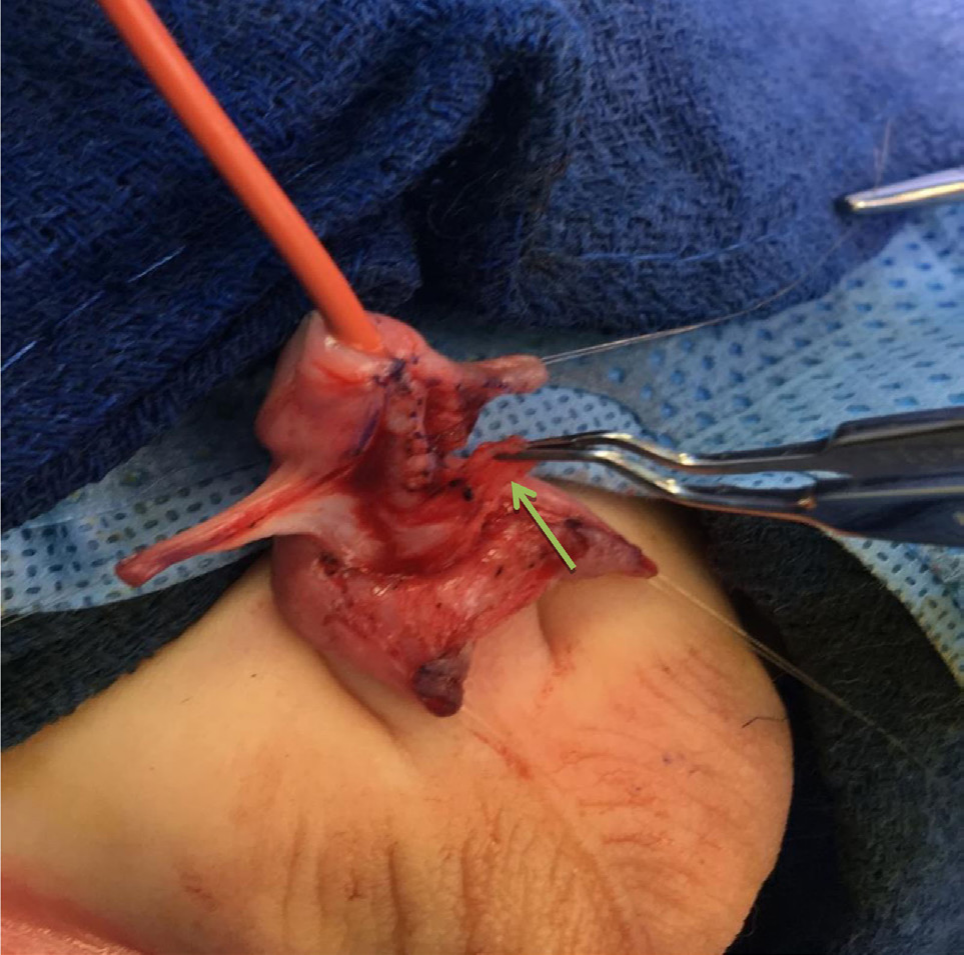
The inframeatal flap has been deepithelialized and is rotated superiorly to cover the reconstructed urethra (green arrow).

Once the urethral plate has been freed up, a small wedge of urethra plate may have to be excised to remove excess mucosa and irregular edges. In addition, as the urethra has been tubularized, the inframeatal flap can be deepithelialized and brought over on to the urethra (Figure 3). Reconstruction of the glans is facilitated by the fact that patients with MIP variant all have a healthy and large glans. The glans wing can therefore be brought over in the midline with little or no tension provided that the dissection of the glans groove was deep.

If the patient is circumcised, the ventral aspect of the penile shaft can be brought back together easily as the glansplasty will narrow the sub coronal gap. If the patient is not circumcised consideration should be given to reconstructing the prepuce. However, attempt to reconstruct the prepuce in hypospadias may be associated with a higher rate of complications specifically dehiscence of the prepuce or secondary phimosis (10). No information was found addressing the issue of reconstructing the prepuce in MIP but as preputial preservation may not always yield satisfactory outcome, our recommendation has been to carry out circumcision at the time of repair.

Glansplasty is facilitated by the pressure of large amounts of glans tissue which can be opposed in the midline. Care should be given to resect some glans mucosa on the medial aspect so on to allow for opposition to be complete. Deep vertical mattress sutures will maintain the glans together. The meatus should be located and matured at the top of the penis. Mucosa of the glans can be brought back together with interrupted stitches of absorbable fine suture. Stenting with a 6 F urethral catheter is recommended for 5–7 days.

A lightly compressive dressing is left in place for 48 h after which petroleum jelly is applied four to five times a day.

## Current Series

A retrospective review of all cases of hypospadias repairs (481 cases) was carried out at our institution from 2007 to 2017 yielded 25 cases of MIP variant hypospadias (5%) who underwent surgical correction. Another seven cases were also evaluated and felt not to require surgical repair given a minor dilation of the urethral meatus and a location close to the tip of the glans.

The 25 patients who underwent surgical repair were divided into two groups. The first 10 patients (group A) underwent a simple tubularization of the urethral plate. In the course of the dissection of the urethral plate, an injury to the lateral aspect of the urethral plate necessitating repair prior to urethral tubularization was noted in six cases. One of these was later found to have a small fistula at the coronal margin which required a repair a year later. The last 15 cases (2010–2017) (group B) underwent repair with a modification of the Mathieu technique using a vascularized inframeatal flap. No urethral injuries were noted as good visualization of the lateral aspects of the urethral plate on either side was provided by using traction on the inframeatal flap. None of these patients experienced subsequent fistula formation. All patients had an indwelling urethral catheter in place for 5–7 days postoperatively. In group B, one patient was found to have very minor glans dehiscence but no other issues and all were reported to have a normal urinary stream. Follow-up was at least 1 year for all patients.

## Discussion

The challenges of repairing the MIP variant of hypospadias were well laid out by Duckett and Keating in their seminal article (2). The first challenge is to identify those patients who truly need surgical repair. In our series, the features identified as important in the decision making process were (1) meatal opening close to or below the coronal margin; (2) A deep glans cleft; (3) a wide, splayed out glans; and (4) an abnormal urinary stream if witnessed.

For those who display these features, surgical repair should provide satisfactory outcome as documented by several series (2–6). In the series presented herein, a retrospective accounting shows that 25 of the 481 hypospadias repairs carried out over the last 10 years had the diagnosis of MIP variant of hypospadias (5%). Surgical outcomes include: (1) normal conical appearance of the glans, (2) a urethral meatus of normal caliber, and (3) a single, normal urinary stream with no symptoms.

Nevertheless, the anatomy of the urethra may be complex in MIP and reconstruction of the urethra may be quite challenging if the anatomy has not been well identified and marked out. Despite a large amount of urethral plate tissue, inadvertent injury to the lateral aspect of the plate can occur in the area where the urethra widens out at the level of the coronal margin. This will compromise repair of the urethra. The technique described above allows for careful identification of the surgical planes and should prevent injury to the urethral plate. This technique will also provide deepithelized tissue that can then be used to cover the reconstructed urethra as advocated by Belman (11). Such coverage with a flap of vascularized tissue should reduce the risk of urethra-cutaneous fistula formation especially in the area of the corona where the tissue coverage is at its thinnest.

The outcomes in our cohort of patients with MIP were in general good but long-term follow-up (past 3 years) was not possible either because patients were too young or have not come back for reevaluation. The one case of urethra-cutaneous fistula occurred within 6 months after repair in a patient who did not seem to have had additional vascularized tissue placed at the time of urethral reconstruction. The small numbers of patient preclude any statistical analysis so no clear conclusion can be made from the observation.

From a technical stand-point, the Mathieu modification or use of the inframeatal flap provides three clear advantages: (1) it allows for better delineation of the urethral anatomy and lateral boundaries. (2) The dissection of the urethral plate is thus made safer by reducing the risk of inadvertent injury. (3) The addition of a layer of vascularized, subcutaneous tissue should reduce the risk of urethra-cutaneous fistula.

Finally, it should be emphasized that prior circumcision does not impact on the subsequent repair of this form of hypospadias. Snodgrass, in a retrospective review of 63 patients divided two groups, those with and without circumcision, reported that circumcised status did not seemed to be associated with a higher incidence of complication (12). This was confirmed by Pieretti et al. (13). In those patients who have not been circumcised, the option of retaining the foreskin should be discussed but, in light of the increased potential complications such as phimosis, circumcision may be the safest approach.

## Conclusion

Megameatus intact prepuce variant of hypospadias is relatively uncommon and can pose a surgical challenge as the margins of the urethra can vary from case to case. Using an adapted Mathieu technique, one can improve visualization of the urethra during dissection and also provide healthy vascularized coverage to the reconstructed urethra. Complications are rare and functional outcomes should be, in general, satisfactory.

## Author Contributions

This is a retrospective review of patients treated for megameatus intact prepuce variant of hypospadias over the last 10 years. A new technique is presented and a review of the literature is provided. I have collected the data and wrote the manuscript.

## Conflict of Interest Statement

The author declares that the research was conducted in the absence of any commercial or financial relationships that could be construed as a potential conflict of interest.
